# Study protocol for a feasibility evaluation of *Charge Up!:* an adaptation of Critical Time Intervention for young adults moving from homelessness to housing

**DOI:** 10.1186/s40814-025-01677-7

**Published:** 2025-07-02

**Authors:** Sarah C. Narendorf, Michelle R. Munson, Umaira Khan, Marcus Brown, Gregory Gomez, Diane Santa Maria, Todd Gilmer, Maurice N. Gattis, Prince Hayward, Daniel Herman

**Affiliations:** 1 New York University, Silver School of Social Work, 1 Washington Square North, New York, NY 10003 USA; 2 University of Houston, Graduate College of Social Work, Houston, TX 3511 Cullen Blvd, 77204 USA; 3 University of Texas Health Sciences Center, Cizik School of Nursing, Houston, TX 6901 Bertner Ave, 77030 USA; 4 University of California, San Diego, Wertheim School of Public Health & Human Longevity Science, 9500 Gilman Drive, La Jolla, CA 92093 USA; 5 Virginia Commonwealth University, School of Social Work, 1000 Floyd Avenue, Box 842027, Richmond, VA 23284 USA; 6 Hunter College, Silberman School of Social Work, 2180 Third Avenue, New York, NY 10035 USA

**Keywords:** Young adult, Homelessness, Rapid rehousing, Mental health, Intervention

## Abstract

**Background:**

Young adults experiencing homelessness (YAEH) have high rates of mental health challenges, yet low rates of mental health service utilization. The transition from homelessness to housing is a key time for intervention to connect YAEH with mental health treatment and provide support to improve both mental health and housing stability. *Charge Up!* is a 6-month, phased support intervention that utilizes a team-based approach to connect young adults to community and mental health support. It is an adaptation of Critical Time Intervention that integrates components of Cornerstone, a mental health support intervention designed for young adults.

**Methods:**

This pilot study uses a phased open trial that begins with a feasibility trial to refine the adapted *Charge Up!* intervention (*n* = 8), then further tests feasibility, acceptability, and preliminary signal of impact in a small, randomized pilot trial (*n* = 52). Participants are young adults moving into a transitional housing to rapid rehousing program (TH/RRH) in Houston, Texas. Quantitative interviews are conducted at baseline, 3 months, 6 months, and 12 months. Qualitative interviews are also conducted at 6 months. Exploratory analyses will examine the feasibility of implementing *Charge Up!*, the performance of measures, and whether *Charge Up!* is changing the hypothesized targets. Qualitative analysis from interviews will examine demand for the intervention, acceptability of the intervention, and integration of the intervention within the housing system context.

**Discussion:**

The aim of this study is to pilot test *Charge Up!*, an adapted version of Critical Time Intervention designed to provide targeted support for mental health at the point of transition from homelessness to housing. The *Charge Up!* intervention was co-developed with young adults and providers, and this phased open trial will help to refine and provide preliminary evidence of the feasibility and acceptability of the intervention. The program is provided in conjunction with a widely used housing model, RRH, and has the potential for scalability as an adjunctive intervention to support youth transitioning into RRH across the United States.

**Trial registration:**

This study was registered on ClinicalTrials.gov on October 20, 2023 (Identifier: NCT06102850), as Protocol ID R34MH129542-01A1, University of Houston, Title: Adaptation of Critical Time Intervention for Young Adults with MH Challenges (CTI-YAMH).

## Introduction

Young adult homelessness is an urgent public health issue with over 3 million young adults experiencing housing instability each year [1]. Over half of young adults experiencing homelessness (YAEH) are impacted by a mental disorder [2], highlighting the urgency of developing interventions to support mental health during the stressful transition from homelessness to housing. Supported housing programs that are funded through the Department of Housing and Urban Development (HUD), such as rapid rehousing (RRH), come with case management support but lack clear guidelines or evidence-based interventions for providing mental health supports in conjunction with housing programming, particularly for young adults. This is becoming increasingly urgent as levels of depression and anxiety have risen in young adults over the last decade [3], particularly in the wake of the COVID-19 pandemic [4].

Prevalence of mental illness is high and growing among young adults. 3.3 million young adults experienced a serious mental illness in 2020, more than double the number reported in 2008 [3]. Young adults that experience housing instability are particularly impacted by mental health problems. Studies examining the prevalence of mental health symptoms have generally found that between 49 and 98% of YAEH meet criteria for a mental disorder [2, 5–7], with 36–67% meeting criteria for two or more diagnoses [2, 7], particularly co-occurring substance use disorders and internalizing disorders such as post-traumatic stress disorder (PTSD) and major depression [7]. About 20–30% of YAEH across studies met criteria for a mood disorder (depression or bipolar) and 5% to 48% met criteria for PTSD [2, 6–8]. This has dire consequences, with YAEH having mortality rates ten times higher than their peers in the general population, primarily due to suicide and drug overdose [9].

YAEH disproportionately come from marginalized groups, highlighting the need to tailor interventions specifically for their needs. In their national household survey of homelessness, Morton et. al. (2018) found that 3.5 million young adults experienced a form of homelessness in the past 12 months and that Black, Hispanic, and Lesbian, Gay, Bisexual, Transgender, and Queer (LGBTQ +) youth were disproportionately affected [1]. These intersecting identities have impacts on mental health and the culturally relevant services needed for treatment [10, 11]. Recent work examining over 1000 YAEH across 7 cities found that experiences of discrimination were widely reported among YAEH and associated with greater psychological distress [12]. Yet, marginalized youth of color, particularly young men, are least likely to seek and receive mental health services [13].

Mental health and housing are closely related. Mental health is fundamentally tied to housing in a bi-directional relationship [14, 15]. Mental health symptoms heighten risk for homelessness in young adults and the experience of being homeless can exacerbate mental health symptoms or trigger onset [14]. Housing instability also contributes to substance use and victimization, circumstances that are linked to mental illness [16, 17]. Conversely, research has shown that housing improves mental health, making it a critical component to providing mental health treatment that has the potential to support long-term housing stability [15, 16]. The period of moving from homelessness to housing has been identified as a “critical time” for providing supports to improve mental health [18]. This may be particularly true in young adulthood, a period where many experience the onset of mental health symptoms [19], symptoms that can be exacerbated by housing instability.

## Service connection and treatment engagement

Young adults are less likely to utilize mental health services than both adolescents and older adults [3] and there is a precipitous drop in service use as youth take charge of their own care at age 18 [20]. YAEH are particularly impacted, with only one third getting mental health services in the previous year [21] despite demonstrated high need [2]. Barriers to service utilization are both perceptual and logistical. Among YAEH who screened positive for a mental health problem, 65% did not perceive that they had any mental health concerns, highlighting the need for developmental interventions that increase awareness and decrease stigma [22]. In addition, YAEH face barriers to successfully engaging in services even when they do identify the need, including uncertainty about where to go, transportation barriers, cost of care, lack of child care, and lack of flexibility in the hours and processes of providers [23]. The point of transition from transience to housing stability presents an opportunity to identify mental health needs and establish new connections to mental health services that can be supported and sustained over time.

Even when young adults receive services, however, they have high rates of treatment dropout. In one study following psychiatric hospital discharge, 50% of young adults did not follow up with treatment, and being African American was associated with increased risk of non-follow-up [24]. Barriers to treatment engagement specific to young adults have been identified and can be targeted to increase engagement [25]. These include negative beliefs or stigma of mental illness, fear, hopelessness, ambivalence, and self-image considerations [26, 27]. Prior research has developed and tested interventions to target these mechanisms, with emerging evidence of success [26, 27]. Specifically, young adults that participated in a group-based engagement intervention demonstrated greater treatment engagement (Cohen’s *d* = 0.59), decreased stigma (Cohen’s *d* = 0.47), increased trust in providers (Cohen’s* d* = 0.51), and more positive beliefs about mental health services (Cohen’s *d* = 0.57) compared to youth that received treatment as usual [28]. Connections to mental health services need to be paired with engagement interventions for young adults to achieve the benefits of treatment.

## Housing first approaches

Housing first approaches can be beneficial for stabilizing mental health and housing. The housing first model has emerged as the standard intervention approach for providing housing to adults with serious mental illness [30, 31]. This unconditional approach to housing has been less well studied in young adults but a randomized controlled trial for adults in Canada found that younger adults had different housing needs than older adults [32]. Compared to older adults in the trial, young adults entered with higher rates of substance use disorders, exposure to victimization, greater unmet needs for health care, and lower educational attainment [32]. These studies highlight the need to think about young adults as a specific population in need of tailored housing supports.

Rapid Rehousing (RRH) is a housing support model that provides housing subsidies paired with case management with the aim of stabilizing housing and providing service connections in a time limited fashion [33]. Across programs, this model provides housing navigation, subsidized rent for a discrete period, and case management [33]. But, there is little consistency across programs in the supportive services provided. Communities across the country have developed a range of approaches to supporting young adults in these programs but few have focused specifically on mental health treatment and engagement in the context of RRH [34–37]. Most recently, in 2017, HUD introduced a new housing model specifically for young adults that pairs more supported, structured housing delivered in a transitional housing program with rapid rehousing, giving young adults more flexibility to gradually transition to the less structured format of RRH [38]. The current study seeks to test an adapted version of the evidence-based Critical Time Intervention [39] for YAEH making a transition from homelessness to supportive housing in a time-limited housing program model such as TH/RRH. We integrated components of an evidence supported intervention for young adults with mental health challenges, Cornerstone Coordinated Community Care (C4) [29] to develop the adapted intervention called *Charge Up!*.

## Building on interventions with evidence in other populations

Critical Time Intervention (CTI) is a phased care coordination intervention that aims to connect individuals to formal and informal supports at a point of transition [39]. CTI identifies targeted time-limited intervention applied at critical transition points as a mechanism for enhancing the efficacy of long-term supports for persons with SMI who are living in the community [18]. CTI has been found to improve a range of outcomes for racially diverse adults with severe mental illness, including preventing homelessness, rehospitalization, and substance misuse after psychiatric hospital discharge [40–42]. CTI is delivered in three phases with decreasing intensity of contact at each phase. The first phase provides the most intensive support as the individual makes the transition that is the focus of the intervention. The focus in this phase is connecting the individual with a broad range of formal and informal support, including long-term supports that can remain in place after the intervention. In the second phase, the CTI case manager continues to provide support but encourages the person to solve problems with the assistance of their support network. In the third phase, the CTI case manager is intentionally stepping back and transferring responsibility to the individual and the long-term supports in their network [39].

Cornerstone Coordinated Community Care (C4) is specifically designed to increase engagement in mental health care and decrease mental health symptoms among adolescents and young adults [29, 43]. It is a psychosocial intervention that combines trauma-focused cognitive behavioral therapy, skills groups, Critical Time Intervention (CTI), and peer support providers called Recovery Role Models to serve youth with mental health challenges [29]. C4 primarily is designed to address engagement in treatment through impacting key targets (i.e., stigma, hope) and mental health symptoms and functional outcomes. The dual provider team of a licensed clinician and a peer provider works both in the clinic and in the community providing case management (i.e., CTI), in vivo supports, therapy and medication management, and skills groups. C4 was evaluated in a small-scale, two-group RCT using a pre-test/post-test design focused on engagement, symptoms, and functional outcomes. The study showed promising results related to decreases in symptoms, improved recovery, along with high feasibility and acceptability in a racially diverse sample of young adults in New York City [44].

## Guiding theoretical framework

**Fig. 1:**
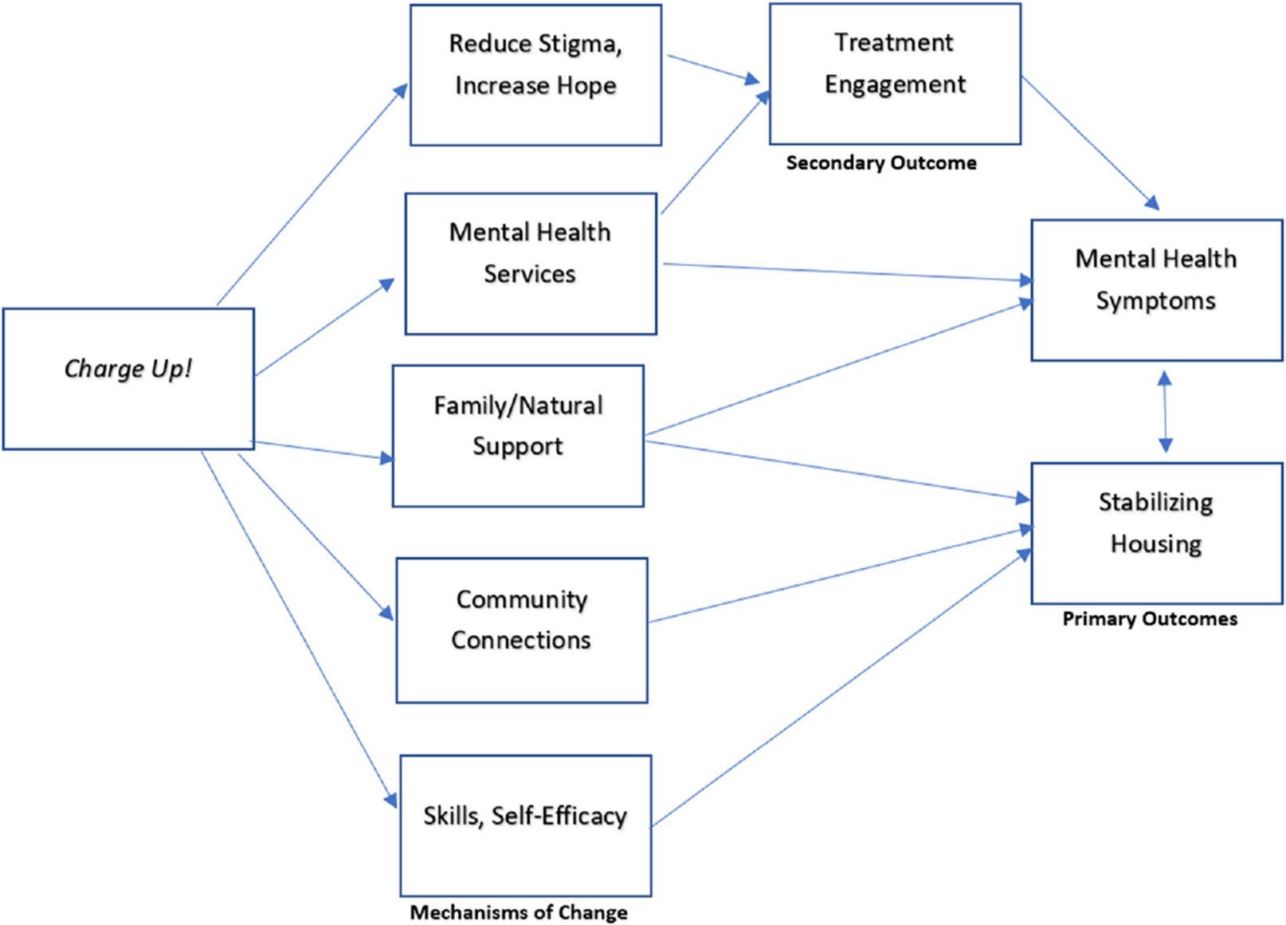
Target framework for adapted charge up intervention

Prior research has identified that interventions for YAEH need to provide an array of supportive services to accompany housing models [45], yet it is currently unclear the types and models of supportive services that may be most beneficial. We began with CTI as a starting point for intervention adaptation. In prior RCTs, CTI has been found to be effective for stabilizing housing in adults with serious mental illness, with some evidence that the hypothesized mediators of connections to family [46] and increased continuity of care [41] are critical targets for success. For young adults with mental health problems who are often not currently in treatment, connection to mental health services paired with support to ensure engagement in treatment are critical. Building on evidence from Cornerstone, *Charge Up!* integrates activities to enhance treatment engagement and connections to services including individual sessions in the community with a peer mentor and addressing socio-emotional needs with a mental health professional. Figure 1 provides the target framework which informs the *Charge Up!* intervention and the measurement of primary outcomes of mental health symptoms and housing stability, the secondary outcome of treatment engagement, and the assessment of whether the intervention is effectively addressing the hypothesized mechanisms of change – reduced stigma, increased hope, connections to services (including mental health services), community connections, family and other natural supports, and self-efficacy.

## The current study

This study aims to rigorously refine and conduct feasibility testing of a newly adapted version of CTI for young adults moving into supported housing that was developed by our team, entitled *Charge Up!* (originally called CTI-YAMH). *Charge Up!* adapts CTI for the specific context of transitioning from homelessness to TH/RRH for young adults and integrates the team approach and the mental health specific content from Cornerstone Coordinated Community Care (C4). The study embeds experimental therapeutics [47], focusing on exploring change mechanisms which have been targeted by CTI and C4 and that have been associated with improved treatment engagement, mental health, and housing stability in prior work. Experimental therapeutics which have been referred to as the science of “how” places focus not just on the impact of the intervention on the primary outcomes but also specifying the specific mechanisms that are hypothesized to result in the change and to rigorously examine and test which of these mechanisms is changing as a result of the intervention and whether that change is ultimately related to change in the primary outcomes.

The aim of this study is to conduct a phased open feasibility trial of the adapted *Charge Up!* Intervention, first refining the intervention (*n* = 8), and then testing it in a small randomized trial (*n* = 52) to assess acceptability, assess the feasibility of the intervention and randomization procedures, refine outcome measures, and conduct exploratory analyses to examine the preliminary signal of impact of the intervention. Through this work, we aim to assess the feasibility and acceptability of the intervention and explore whether it shows promise for improving our proposed mediating targets as well as our proposed primary and secondary outcomes—treatment engagement, housing stability and mental health symptoms. We also aim to better understand the feasibility challenges of conducting a randomized trial within the context of an existing housing interventions.

## Methods

### Study Design

The study employs a phased, open trial to first refine the intervention and procedures, then further test feasibility in a small randomized controlled trial of the refined intervention (see Table 1). The design of this feasibility pilot will enable us to examine several issues that we anticipate will need refinement prior to larger scale testing. First, we aim to understand the feasibility and acceptability of the intervention within the context of an existing housing intervention program. Through our first phase, we will focus on assessing the feasibility and acceptability of the proposed intervention, focusing specifically on refining procedures for team coordination and communication, documentation protocols, and developing fidelity measures. We will also examine commonly identified assessment targets for feasibility studies [48], including acceptability, demand for the intervention, practicality of the intervention, and feasibility of integration into the current housing system.

**Table 1:** Randomized feasibility trial of Enhanced Housing Support Intervention, Charge Up! (*n* = 52)

	STUDY PERIOD							
	Enrollment	Allocation	Post-allocation				Close-out	
TIMEPOINT**	*-t*_*1*_	0	*t*_*1*_ _baseline_	*t*_*2*_ _3 month_	*t*_*3*_ _6 month_	*t*_*4*_ _12 month_		*t*_*x*_
Enrollment:								
Eligibility screen	X							
Informed consent	X							
Allocation		X						
Interventions:								
*Charge Up!*								
*Housing support as usual*								
Assessments:								
Mental health diagnoses	X					X		X
Mediating targets			X	X	X	X		X
Mental health symptoms and housing stability			X	X	X	X		X
Fidelity assessment				X	X			X

We will also assess aspects of the research methods and measures in preparation for conducting a larger randomized trial. The first key issue is randomization. Because the *Charge Up!* intervention is implemented for young adults with mental health issues who are transitioning from homelessness to supported housing in the TH/RRH program, randomization needs to occur at the point of assignment to an agency for TH/RRH. The primary agency who partnered with us to develop the intervention, is one of several agencies in the area that provides TH/RRH services for young adults. Because randomization is happening in the context of assignment to services, we need to determine the optimal time to screen and enroll participants in the study so that there is the ability to randomize when a spot becomes available. Second, we will assess and document the suitability of the control condition, treatment as usual (TAU), which is conducted system wide at agencies that provide young adult TH/RRH. There is variation in how TH/RRH is implemented across agencies in the community in the length and setting (scattered vs congregate) for the program.. A third key issue of the feasibility pilot is the refinement of measures. Measures of our mediators were selected based on CTI research in adult populations and may need refinement for young adults, so we will examine multiple measures for several constructs, examining internal consistency with Cronbach’s alpha. We will also explore additional ways to measure several mediators beyond self-report through documentation procedures of team members and administrative data that can provide additional measures of the constructs. We will also conduct exploratory analyses using correlations to see whether our measures are related to each other as we hypothesize they will be. Finally, we will conduct process mapping and team-based costing activities to estimate the costs of the intervention. Together, examination of these specific areas will provide important information that will prepare us to conduct rigorous testing of the intervention in a future larger randomized trial.

### Sample

Our sample will include young adults (age 18–24) who have qualified for TH/RRH supports, an eligibility determination that happens based on their score on a vulnerability index administered when they are placed on the housing waitlist. Young adults are assigned to a case manager based on capacity across agencies that provide TH/RRH. For phase 1 of the feasibility trial (*n* = 8), we will work with our partner agency to enroll young adults as they transition into the TH/RRH program. Referrals for this first phase will be provided by our partner agency and we will work closely with the case managers at this agency to refine our procedures and refine the intervention as we learn during this first phase. We will also use this first phase to refine the measures for the study. Structured interviews will be administered by a trained member of the research team at baseline, 3 months, 6 months, and 12 months.

For the second phase of the study, we plan to expand the intervention to be available at any of the three agencies in the community that offer TH/RRH. We will identify young adults who qualify for the *Charge Up!* intervention in conjunction with their prioritization for housing. The screening tool used to prioritize assignment to housing asks for a self-report of lifetime mental health diagnoses, and we will add the Kessler-6 to screen for current mental health problems. Those that report prior mental health diagnosis or screen positive for current symptoms will be eligible for *Charge Up!* and will be provided with information about the study. Those that are interested will meet with a member of the research team for an intake and participate in a baseline interview. The intake packet will contain a card which has information about randomization prepared using a random numbers table, which will be opened following the initial assessment. We will closely track the feasibility of this process, keeping track of the number of YAEH who are on the wait list, the number contacted, the number successfully enrolled, and the number that meet our enrollment criteria.

For the first phase of the open trial, we aim to recruit a sample of 8 young adults who are receiving TH/RRH services at our partner agency. During the second phase of the trial, we will expand recruitment to enroll 52 young adults with mental health history or symptoms and randomize half to each condition. Based on current housing capacity and our prior research, we estimate that 60–75% will screen positive for mental health problems as assessed with the Kessler-6 or prior history so at current capacity we would identify 4–6 participants per month that are eligible for the study and enroll 2–3 of these for approximately 12 months in the intervention condition and 2–3 participants per month for 12 months in the TAU condition.

### Eligibility determination and retention

Young adults (age 18–24) will be screened for study inclusion criteria (meeting criteria for psychological distress on Kessler-6 [49] or self-reported prior diagnosis of depression, anxiety, bipolar or a psychotic disorder) and invited to participate at the point of assignment to a TH/RRH program. Young adults will be told of the opportunity to participate in the study at the time of intake to a program and asked for permission to provide their names to the study team if interested. When the young adult meets the centralized housing criteria that qualifies them for TH/RRH, they are referred to a receiving agency who will provide names to the study team. The team will screen for eligibility and schedule a baseline interview. Trained graduate-level research assistants from the study team will obtain informed consent and administer the survey in person or via phone or Zoom. In order to track outcomes through the course of the 6-month intervention, we will administer surveys at baseline, the midpoint (3 months), end of the intervention (6 months) and follow-up 6 months later (12 months). We will also conduct qualitative interviews with all young adults in the first phase of the feasibility pilot (*n* = 8) and each of the professionals involved in implementing it (*n* = 5) to explore qualitatively assess implementation outcomes of acceptability and appropriateness. Interviewers will contact all young adults in both conditions of the trial monthly to maintain contact and increase retention. At the time of enrollment, a locator form will be completed to gather detailed data about contact information including social media contacts and the names of others who may know how to contact the young adult. Participants’ incentives for completing study interviews increase over time with the aim of increasing retention.

### Setting and location

This study will take place in Harris County, TX, which contains the city of Houston. In Fall 2021, the community was awarded 10 million dollars through a Youth Homelessness Demonstration Project (YHDP) Grant from Housing and Urban Development (HUD) which funded three TH/RRH providers in the county. Entry into the homelessness system is managed through a coordinated access system with a consistent assessment process and centralized wait list, which is prioritized based on a screener that asks respondents to self-report whether they have been diagnosed with a mental disorder. For the first phase of this study, we will partner with one community agency that runs a TH/RRH program and has on-site housing provided by the agency. We plan to potentially expand to include two other community agencies that also provide TH/RRH for the second phase of the pilot, where we randomize young adults to either receive the intervention or housing supports as usual. These agencies provide both scattered site housing as well as on-site housing. TH/RRH is a newer housing model that HUD approved for funding in 2017. It is specifically designed for young adults to allow them to spend some time in a more structured setting (transitional housing) prior to transitioning to their own apartment in rapid rehousing (RRH) where they are expected to pay a portion of their rent. The programs are offered together but allow young adults some flexibility in whether they desire to start in TH or go directly to RRH, and also whether they desire to transition to RRH as the plan after TH. The community setting for this study is in a learning phase for offering this combination of housing, so expanding to three sites could allow us to examine the feasibility of *Charge Up!* across multiple agencies who may be managing the program in somewhat different ways.

### Description of the Charge Up! intervention

Based on the results of our preliminary work, we identified three roles that we have incorporated into the drafted manuals for refinement (summarized in Table 2). The content of the intervention was developed by adapting CTI with components of Cornerstone through a stakeholder feedback process that centered a young adult working group (YWG). The YWG led the development of a set of guiding values and provided feedback on the roles for each member of the *Charge Up!* team. Our primary partner agency that will collaborate on the first phase of the trial, was trained on the intervention prior to the start of the first phase of the trial. Prior to the start of phase 2 of the intervention, the TH/RRH case managers will be trained in the refined *Charge Up!* intervention. Two new roles will be added to support the case managers to provide enhanced mental health supports which are funded through the study. A mental health professional called a healing partner will assess, provide time limited mental health support, and ensure connection to mental health treatment when a young adult desires it. A transition support specialist (adapted from the recovery role model in Cornerstone Coordinated Community Care, C4) will also be part of the team. This will be a person with lived expertise of mental illness and housing instability who has been successful in achieving housing stability and successfully managing their mental health for at least a year. This role provides coaching and skill building activities and instills hope, providing a model of success. The intervention will run for 6 months, the time period proposed for CTI for RRH with adults, though case managers remain involved with young adults in a limited role as long as they are receiving RRH (average of 12 months).

**Table 2:** Charge Up! intervention roles adapted from prior interventions

Core component	Lead personnel	Difference from current practice	Original intervention
Transitional case management based on CTI model	TH/RRH case manager	Training in CTI approach for current case managers	CTI C4 CTI elements
Mental health assessment and treatment connections	Healing partner	Currently referred out, now starts in the Charge Up program, with connection to longer term supports	C4 boundary spanning case manager
Mentoring and skill building	Transition support specialist	Currently no structured skill building activities or mentorship	C4 recovery role model

Treatment as usual will be assignment to one of the community agencies administering the TH/RRH program with housing and case management alone. Because TH/RRH is a developing program in our setting, we are prepared to carefully track TAU across multiple agencies. All TH/RRH programs currently provide the same housing support model, which provides time-limited assistance with housing and a housing case manager; however, the length of TH and RRH varies across agencies. Referral resources for mental health treatment will be provided at the point of intake for all that screen into the study but are randomized to TAU. We will assess the frequency of contacts with the case manager and other supports provided in TAU through structured items during interviews at 3, 6, and 12 months and include open-ended questions to describe supports provided.

### Feasibility and acceptability measures

To assess feasibility outcomes we will examine the constructs of acceptability, demand, practicality, and integration [48]. To assess demand, we will track how many YAEH are referred to the intervention, how many go through screening and express interest in the study, and how many are successfully enrolled. We will track how many sessions with intervention team members youth attend (in person or via zoom) at each phase of the intervention and our team will track retention of intervention and control condition participants at each measurement point, implementing strategies to increase retention if needed. Acceptability will be assessed through qualitative feedback about the intervention through open ended questions at each measurement point and as part of the final qualitative interview with participants and case managers. Practicality and integration will be assessed through qualitative interviews with case managers at the end of each phase of the study through questions about the challenges of coordinating with the larger team, the time involved in supporting the intervention, and its fit with the existing set of referral processes and resources available in the system.

### Proposed outcome measures

A summary of measures identified to assess proposed outcomes in the study is provided in Table 3. Measures of intervention targets used in prior studies of CTI with adults are included as well as measures used previously in C4 to assess stigma and hope. In addition, case managers will report on community connectedness, social support, and family relationships to provide additional data to triangulate and strengthen the self-report measures. The proposed measures will be assessed for acceptability based on length of the interview, participant response burden, and interviewer feedback following the first phase of the feasibility study and then used to explore reliability using Cronbach’s alpha following the randomized feasibility pilot.

**Table 3:** Proposed outcome measures for Charge Up!

Construct	Instrument	Reliability/validity	Timing
Primary outcomes			
Housing stability	Housing Security Scale [50] Number of days homeless, Number of moves in prior 90 days	Alpha = 0.68; housing satisfaction subscale alpha = 0.74 [50]	Baseline, 3 months, 6 months, 12 months
Mental health			
Diagnosis of common disorders	MINI International Neuropsychiatric Interview Screen 7.0.2 [51, 52]	Kappa = 0.51–0.84 [48]	Baseline, 12 months
Depressive symptoms	Patient Health Questionnaire; PHQ-9 [53], 8 items	Alpha = 0.86–0.89 [53]	Baseline, 3 months, 6 months, 12 month
Anxiety symptoms	Generalized Anxiety Disorder; GAD-7 [54], 7 items	Alpha = 0.92 [54]	Baseline, 3 months, 6 months, 12 month
PTSD symptoms	Primary Care PTSD Screen (PC-PTSD) [55], 4 items	Test re-test = 0.83, Sensitivity = 0.78, Specificity = 0.87 [55]	Baseline, 3 months, 6 months, 12 months
Other mental health symptoms	Colorado Symptom Inventory [56], 14 items	Alpha = 0.77–0.85 [56]	Baseline, 3 months, 6 months, 12 months
Secondary outcomes			
Mental health service use/treatment engagement	Self-report of taking medication, attending appointments; casemanager report of appointment attendance Client Engagement in Child Protective Services Scale [57], modified for mental health services; 8 items	Strong face validity, used in prior studies Alpha = 0.91 [58]	Baseline, 3 months, 6 months, 12 months Baseline, 3 months, 6 months, 12 months
Hypothesized mediators (underlying mechanisms)			
Family relations	2 subscales Quality of Life Inventory, 3 items [59] Systematic Clinical Outcome Routine Evaluation Index of Family Functioning and Change [60] Case manager report	Alpha = 0.67–0.87 [59] Alpha = 0.90–0.93 [60]	Baseline, 3 months, 6 months, 12 months
Social support	MOS Support Scale, 15 items [61] Interpersonal Support Evaluation, 12 items [62] Case manager report	Alpha = 0.93 [61] Alpha = 0.91 [62]	Baseline, 3 months, 6 months, 12 months
Community connectedness	Community Integration Scale [63], 18 items Adult Personal Well Being Index Assessment Scale [65], 7 items Case manager report	Alpha = 0.75 [64] Item loadings = 0.51–0.72 [66]	Baseline, 3 months, 6 months, 12 months
Stigma	Stigma subscale, Inventory of Attitudes Toward Seeking Mental Health Services [67], 12 items	Alpha = 0.83 [68]	Baseline, 3 months, 6 months, 12 months
Hope	The Hope Scale [69], 12 items Life Orientation Test-Revised, 10 items [70]	Alpha = 0.71 Alpha = 0.78 [70]	Baseline, 3 months, 6 months, 12 months
Self-efficacy	Generalized Self-efficacy scale [71], 10 items Perceived Behavioral Control [73], 8 items	Alpha = 0.86–0.94 [72] Alpha = 0.60, 0.78	Baseline, 3 months, 6 months, 12 months

### Treatment fidelity

Treatment fidelity in the CTI intervention is assessed through the use of standardized documentation that tracks the phases and contacts for each participant [74]. We have adapted those forms for this study and will assess how well they work for *Charge Up!* and refine them through the first phase of the trial. We have identified guiding principles with our youth working group for the *Charge Up!* intervention (Grace, Authenticity, Accountability, Power Sharing, Systems Change and Culture of Care) and we will focus on incorporating questions into the documentation and/or developing new tools that operationalize these principles in action across activities. We will iteratively test and modify fidelity measures to create documentation to assess fidelity that will accompany the *Charge Up!* intervention at the end of this study.

### Data management and analysis

Qualitative data will be digitally recorded, then transcribed and stored on a secure data platform. Quantitative data will be collected using Qualtrics and stored on the secure data platform. Participant data will have a study participant number but no other identifying information and will be stored on the secure platform in a password protected file separate from identifying information. Structured interview data will be reviewed regularly by the study team for completeness and examined for missing values or outliers.

For the qualitative analysis focused on feasibility and acceptability of the *Charge Up*! intervention, we will analyze open-ended responses from participants at each survey administration and transcripts of interviews with participants and intervention staff using thematic content analyses [75]. A team of coders will develop a codebook through iterative discussions, then code interviews and examine common themes across the sample [76]. Preliminary results will be shared with participants (YAEH and staff) in a member checking process and compared with the quantitative results on the implementation outcomes measures. Intervention procedures and study measures will be modified based on feedback received in the first phase of the feasibility study for further testing and exploration in the randomized feasibility pilot in phase 2.

#### Progression criteria

Following the conclusion of the second phase pilot and feasibility trial, we will assess several criteria to determine whether the intervention and associated procedures are ready for progressing to a larger trial. These are presented in Table4 below in line with prior recommendations [77] to clearly delineate the key considerations that would indicate that the trial is not successful and procedures need to be reconsidered (STOP), criteria that would suggest the need to amend current procedures but proceed with the next phase (AMEND) and criteria that would indicate success without need for significant refinement (PROCEED).

**Table 4:** Progression Criteria Following Phase 2 Trial

	STOP	AMEND	PROCEED
Recruitment	<50% of participants approached agree to randomization and are enrolled in the study	50–75% of participants approached agree to randomization and are enrolled in the study	>75% of participants recruited are enrolled
Intervention Delivery	<50% of the study participants randomized to the intervention are still receiving the intervention 3 months after enrollment	50–75% of the study participants randomized to the intervention are still receiving the intervention 3 months after enrollment	75% or more of study participants randomized to the intervention are still receiving the intervention 3 months after enrollment
Acceptability	More than half of staff and participants report problems with acceptability, minimal clear targets identified for improvement	Most participants and staff report intervention is acceptable and there are clear targets for improving staff and participant experience	Intervention participants and staff report intervention is acceptable to them
Study Retention	<50% of those enrolled in the study complete the 6 month follow up interview	50–70% of those enrolled in the study complete the 6 month follow up interview	70% or more of the study participants complete the 6 month follow up interview

#### Sample size and quantitative analysis

The quantitative data analysis will focus on examining recruitment enrollment patterns across the study, retention across measurement points, ensuring measures are appropriate, and estimating initial costs. The sample size of *n* = 52 for the pilot RCT was primarily chosen for pragmatic reasons based on the feasibility of study recruitment procedures [78]. We projected that 4–6 participants would meet criteria and be enrolled each month over a 1-year period. The objective of this pilot study is primarily to determine feasibility of recruitment and randomization procedures and estimate retention, not to estimate effect sizes. For our primary objective of study retention of 70%, our sample size of *N* = 52 will be sufficient to estimate this within a 95% confidence interval from 57.6 to 82.4%. We will also examine the performance of measures in a descriptive fashion to explore their acceptability and performance in the study population. Thus, while the sample size will not permit factor analytic methods, reliability and univariate item-level statistics and intercorrelations will be explored. Based on confidence intervals described by Hertzog [79], with optimal retention, our sample of 52 with 26 per group allows for estimation of Cronbach’s alpha as low as 0.70 (0.59, 0.81) with 90% confidence intervals. We will also conduct exploratory analysis of correlations between hypothesized mediators and the intervention and between hypothesized mediators and the primary outcomes. As described by Herzog [79], our optimal sample of 26 per group will enable estimation of correlations at *r* = 0.30 (0.07, 0.50) with 90% confidence intervals. These analyses are all exploratory and intended to inform design of a subsequent trial rather than establish effect sizes or draw conclusions about measure reliability or relationships between variables.

Costing activities will estimate the incremental costs of *Charge Up!* from the perspective of the agencies delivering the intervention. The costs will be estimated using a combination of process mapping and time-driven activity-based costing [80]. Process mapping will elucidate the elements involved in the implementation of *Charge Up!*, including agency resources and staff involved in supporting the administration of the intervention. Time-driven activity-based costing will assign levels of effort and costs associated with each activity identified in the process mapping. Costs will be estimated using standard accounting methods and administrative budgets. We will also explore ways of quantifying benefits of *Charge Up!*. These activities will enable us to propose a well-informed cost–benefit analysis for a future larger trial.

#### Ethics and dissemination

This study was approved by the University of Houston Institutional Review Board (IRB), STUDY00003854: Adaptation of CTI for YAMH, on October 7, 2022. We have appointed a Data Safety Monitoring Board (DSMB) to provide feedback on participant safety throughout the research. Any changes to the approved protocol will be reviewed and approved by the IRB and reported to the DSMB. The investigators will carefully monitor adverse events with a standardized tracking log provided by our university institutional review board. The research staff receive four hours of training prior to the start of each phase of the research to identify distress in study participants, protection of human subjects, strategies for interacting with YAEH, collection of accurate data, ethical issues, trauma-informed approaches to interviewing, and maintaining appropriate boundaries. Participants are asked for consent to have their data shared through the NIMH National Data Archive and these data will be uploaded twice a year until the trial is complete.

## Summary and future directions

There are a few limitations that should be considered in relation to this pilot trial. The fact that the *Charge Up!* intervention is delivered in conjunction with existing housing programs introduces variation that may impact our target outcomes of housing stability and improved mental health. While all programs in the community are providing TH/RRH, they vary in the type of housing provided (on-site vs. scattered site) and their flexibility in the length of time they aim to provide housing support. We will collect data and document the impact of the housing program and its rules and supports on outcomes as we conduct the trial. Ideally, randomization will allow us to see whether *Charge Up!* has an effect on our outcomes when comparing young adults who received housing within the same housing program as well as comparing all young adults by condition across housing programs. It is also not possible to blind young adults or interviewers to the intervention condition, which is a potential limitation that could influence reporting of outcomes by young adults and assessment of outcomes by interviewers. We will provide training to interviewers and raise this issue within the training to help mitigate this potential bias.

The point of transition from homelessness into a supported housing program is a critical period for intervention to build skills and connections with long term supports that can improve housing stability and improve mental health. The *Charge Up!* intervention has been designed to provide targeted supports through a team-based model. This study will assist in building preliminary evidence around the feasibility and effectiveness of the intervention. Qualitative and quantitative data will assist in ensuring that the intervention is promising and feasible for future larger trials. If effective, *Charge Up!* provides a model that can be integrated into housing programs, building a team to work in a structured way. Many housing interventions incorporate peer supports and mental health professionals in some way; however, *Charge Up!* may help programs better structure supports that are available but not currently integrated in a targeted and phase-based way. The intervention is timely given the large investment by HUD in ending youth homelessness and investing in RRH and TH/RRH for youth and young adults. *Charge Up!* has the promise of providing a model that can improve housing outcomes for these programs while also improving mental health.

## Data Availability

The datasets generated during the current study will be available in the National Institute of Mental Health, National Data Archive, https://nda.nih.gov/.
